# The Film in Schools

**Published:** 1921-02-26

**Authors:** 


### The Film in Schools.

More and more those who may be concerned with the care of child life and the education of the young are learning to realise the important part which, for good or evil, the cinematograph must play in the history of civilisation. So far as it has gone it has been a means principally of entertainment, and its influence in that respect has been mixed. In some parts of the country it has already

**THE PUBLIC WELL-BEING**—(*continued*).

been claimed by the police that the picture-house has had a definite effect in reducing adult crime in specified areas. It is too early yet to put such claims to a sufficiently wide and decisive test; but there is plenty of common-sense in the argument that the cinema, which is now ubiquitous, has largely taken the place of the public-house for the nightly entertainment of a large portion of the population, that it gives people a new and healthy interest in life and keeps them out of mischief by keeping them out of temptation.

Crime and the Picture-house.

The influence which the picture-house exercises on children is more in doubt. In reply to a question the other day in the House of Commons which voiced that doubt, it was pointed out on behalf of the Home Office that the number of juvenile crimes in this country has diminished since the war and is now smaller than it was in 1914. On the other hand, recent evidence suggests that juvenile crime is more serious in character than it used to be, and we must certainly contest the assertion that the actual reduction in the figures is directly due to the cinema. We believe a good deal of the credit for checking crime must be given partly to the better organisation of the elementary schools and the influence of the teaching given in them, and partly to the more humane and rational system of dealing with juvenile offenders introduced by the Children's Courts. We are in no doubt whatever that a very popular class of film does exercise a harmful effect on the child mind, even though it may not be immediately apparent in vicious actions. The existing and extraordinarily ingenious and "realistic" screen stories which relate the details of suicides, murders, or the adventures of successful "crooks," and present the most lurid form of melodrama, provide meat much too strong for the average child; and we are personally aware of children who, after two hours' absorption of sensational experiences of this kind, have lost their sleep, become moody and restless and difficult to handle, and, in some cases, unnaturally nervous and apprehensive. There is much in the cinema that is wholesome as well as interesting, and the present tendency to give it a greater artistic significance by the dramatisation of classic novels and the representation of first-class plays, as well as the "educational" experiments (for adults) courageously tried in some picture-houses, is a move in the right direction. But it is difficult to see how the characteristics that are dangerous to a child's mental poise will ever be wholly eliminated, and for that reason alone the proposal specially to censor programmes of exhibitions open to children should be welcomed by everybody concerned in child welfare.

Education in Pictures.

On the credit side in this question is to be placed the movement which is going forward for the introduction of the cinema into the schools, and particularly, as a commencement, into the continuation schools, as a direct means of education. Mr. Morley Dainow, who is a member of the Kinema Commission and a lecturer on practical psychology under the L.C.C., pointed out very effectively the other day the far-reaching advantages which may result from the inclusion of the cinematograph in the school curriculum. It is, as he showed, in the presenting of facts and in the help towards gaining sounder ideas that the cinematograph can be made to become increasingly useful. The danger in its use for such purposes is that both scholars and teachers may grow lazy. As a means of instruction, therefore, it should only be used when other means are inferior, and never when other means are superior. But Mr. Dainow says he knows of no other means of illustration which could so efficiently and attractively bring home to adolescent minds the immensity of their tasks in science, industry, commerce and administration. We confess that we have little faith in the pretentious and not always disinterested claims put forward for the cinema as a means of moral and educational instruction for the young when such claims are associated with the ordinary picture-house. That claim is, in our opinion, little more than clever camouflage. Children will not be persuaded to pay twopence or threepence or sixpence to "go to the pictures" to be instructed, and no shrewd management will ever expect or try to get them there on such terms. The one place in which the cinematograph can be made really useful as a means of instruction is in the ordinary school curriculum.

